# Absence of Rgs5 Influences the Spatial and Temporal Fluctuation of Cardiac Repolarization in Mice

**DOI:** 10.3389/fphys.2021.622084

**Published:** 2021-03-18

**Authors:** Zi-liang Song, Yang Liu, Xu Liu, Mu Qin

**Affiliations:** Department of Cardiology, Shanghai Chest Hospital, Shanghai Jiao Tong University, Shanghai, China

**Keywords:** G-protein signaling 5, ventricular arrhythmic, cardiac repolarization, QT variability, spatial dispersion

## Abstract

**Aims:**

This study investigated the contribution of the regulator of G-protein signaling 5 (Rgs5) knockout to the alteration of the action potential duration (APD) restitution and repolarizing dispersion in ventricle.

**Methods and Results:**

The effects of Rgs5^–/–^ were investigated by QT variance (QTv) and heart rate variability analysis of Rgs5^–/–^ mice. Monophasic action potential analysis was investigated in isolated Rgs5^–/–^ heart. Rgs5^–/–^ did not promote ventricular remodeling. The 24-h QTv and QT variability index (QTVI) of the Rgs5^–/–^ mice were higher than those of wild-type (WT) mice (*P* < 0.01). In WT mice, a positive correlation was found between QTv and the standard deviation of all NN intervals (*r* = 0.62; *P* < 0.01), but not in Rgs5^–/–^ mice (*R* = 0.01; *P* > 0.05). The absence of Rgs5 resulted in a significant prolongation of effective refractory period and APD in isolated ventricle. In addition, compared with WT mice, the knockout of Rgs5 significantly deepened the slope of the APD recovery curve at all 10 sites of the heart (*P* < 0.01) and increased the spatial dispersions of S_max_ (COV-S_max_) (WT: 0.28 ± 0.03, Rgs5^–/–^: 0.53 ± 0.08, *P* < 0.01). Compared with WT heart, Rgs5^–/–^ increased the induced S1–S2 interval at all sites of heart and widened the window of vulnerability of ventricular tachyarrhythmia (*P* < 0.05).

**Conclusion:**

Our findings indicate that Rgs5^–/–^ is an important regulator of ventricular tachyarrhythmia in mice by prolonging ventricular repolarization and increasing spatial dispersion in ventricle.

## Introduction

The regulator of G-protein signaling 5 (Rgs5) negatively regulates G-protein-coupled-receptor-mediated signaling by accelerating the activity of GTPase and dissociation of GTP-bound Gα subunit (Zhou et al., 2001). Rgs5 has been demonstrated to be highly expressed in cardiac tissue and display an important role in vessel and cardiac hypertrophy prevention (Li et al., 2010, 2019). Our previous study showed that knockout of Rgs5 prolonged cardiac repolarization through reconstructing voltage-dependent K^+^ currents (Qin et al., 2012), but the electrophysiological mechanisms of arrhythmogenesis need to be better understood.

Ventricular repolarization is a process in which the duration varies with site and internode. The mechanism of ventricular repolarization leading to spatial heterogeneity is mainly related to different distributions of ion channels (Antzelevitch, 2007), and genesis of temporal fluctuation was associated with sympathetic tone (Myles et al., 2008; Hinterseer et al., 2010). In the past decade, several studies have examined dispersion and recovery of epicardial action potential (AP) as a means for quantifying spatial repolarization lability (Coronel et al., 2009; Maoz et al., 2014; Srinivasan et al., 2019). Moreover, the method of analyzing beat-to-beat QT variability has enabled investigators to determine the prognosis of temporal fluctuation of ventricular repolarization (Dobson et al., 2013).

In this study, we experimentally analyzed the effect of Rgs5 knockout to alteration of action potential duration (APD) restitution and repolarization dispersion. Our findings indicated that absence of Rgs5 has a significant impact on APD restitution and repolarization heterogeneity. These results are helpful to further understand the occurrence and development of Rgs5-related ventricular arrhythmia.

## Materials and Methods

### Experimental Animals

Male Rgs5 knockout (Rgs5^–/–^) mice (*n* = 22) of C57BL/6 background (8–10 weeks old) and wild-type (WT) mice (*n* = 22) were provided by the Animal Model Centre of Shanghai Jiao Tong University. All study protocols are in compliance with the Guide for the Care and Use of Laboratory Animals published by the United States National Institutes of Health (revised 2011) and approved by the Animal Care and Use Committee of Shanghai Chest Hospital.

### Transthoracic Echocardiography

All mice were examined with a Sonos 5500 ultrasound (Philips) at ultrasonic frequency of 15 MHz, in the short axis view of left ventricular papillary muscle. Left ventricular end-diastolic diameter, left ventricular end-systolic diameter, fractional shortening (FS), and ejection fraction (EF) were measured from LVM-mode tracing with a sweep speed of 50 mm/s at midpapillary muscle level.

### 24-h Telemetry Electrocardiogram Recording

The mice were anesthetized by intraperitoneal injection of sodium pentobarbital (60 mg/Kg) and placed on a 37°constant temperature heating plate to fix their limbs. The subcutaneous tissue was bluntly separated to form a capsular bag through the right-side incision (1–2 cm) of lower abdomen and then place the body of implant (EA-F20, DSI) into the capsular bag. The anode and cathode electrodes were sutured under the skin of right shoulder and left groin according to standard II lead position. Electrocardiogram (ECG) measurements (lead II) were recorded in Rgs5^–/–^ and WT mice. The ECG amplifier module (data acquisition system [DSI], United States) included low- and high-pass filters (set to 1 kHz and 0.05 Hz, respectively) and a gain selection device (set to 1000-fold). Signals were continuously digitized at a frequency of 1 kHz and recorded using DSI (United States). P3 software provided by the data acquisition system was used to analyze telemetry ECG data. The heart rate (HR) of each mouse was continuously recorded for 24 h and analyzed.

### QTV and HR Variability Analysis

According to the previous study of Zhang et al. (2014), QT intervals were calculated as the time at which the negative T-wave reached to the isoelectric baseline. Isoelectric baselines were determined between the end of the P-wave and the beginning of the QRS. QT mean (QTm) and QT variability (QTv) were calculated in each 2-min segment from 24-h ECG waveforms to assess QT interval variability. The QT variability index (QTVI) was then determined using the formula: QTVI = log_10_[(QT_V_/QT_m_^2^)/(RR_V_/RR_m_^2^)] (Piccirillo et al., 2009). In HRV analysis, the parameters of time domain analysis included the standard deviation of all NN intervals (SDNN, ms), SDNNindex, rMSSD, pNN5, and LF/HF of the light period and the dark period.

### Preparation of Langendorff-Perfused Hearts

The isolated heart was prepared as described in our previous study (Qin et al., 2012). After anesthesia and heparinization, the isolated heart was rapidly resected and transferred to Langendorff perfusion system (ADInstruments Pty Ltd, Australia). The heart was perfused retrogradely through aorta at a rate of 2–2.5 mL/min. In this way, the heart was perfused by HEPES-buffered Tyrode’s solution (KCl, 5.4 mM; NaCl, 130 mM; CaCl_2_, 1.8 mM; Na_2_HPO_4_, 0.3 mM; MgCl_2_, 1 mM; glucose, 10 mM; HEPES, 10 mM; pH adjusted to 7.4 with NaOH) passing through aorta into coronary arteries. The isolated heart was perfused for 20 min before next experimental test. The heart was discarded if irreversible myocardial ischemia occurred or it did not return to normal spontaneous rhythm.

### Monophasic AP Recording

A customized single-phase AP (MAP) electrode is composed of two 0.25 mm Teflon-coated silver wires (purity 99.99%) wound together for recording MAP and electrolysis to eliminate DC offset. The MAP is amplified by an amplifier with a bandpass filter between 0.3 Hz and 1 kHz. Epicardial MAP recordings were performed at right basal ventricle, right ventricular outflow tract, apex and free wall of right ventricular, left posterior and anterior basal ventricle, left posterior and anterior apex and left posterior and anterior free wall. The paired platinum stimulating electrode was positioned on basal surface of right ventricle. At baseline cycle length (CL) of 125 ms, routine pacing stimulation (Grass, United States) was performed with square wave stimulation lasting for 1 ms, with amplitude 3 times diastolic threshold. Chrat7.0 software was used to analyze MAP waveforms.

### Experiment Stimulated Protocol

The effective refractory period (ERP) was measured by programmed electrical stimulation (PES) in different parts of the heart, Left ventricular anterior base (LAB), left ventricle anterior middle wall (LAM), left ventricle posterior base (LPB), left atrial appendage (LAA), left ventricle posterior middle wall(LPM), left ventricular posterior apex(LPA), right ventricular outflow tract(RVOT),right ventricular base(RB), right ventricular middle wall (RM), and right ventricular apex (RA). For PES consisting of 8 stimuli (S1) and 9th extra stimuli (S2), CL of S1 sequence was < 125 ms. The first S1–S2 interval was equal to pacing interval and then gradually decreased until S2 stimulation no longer caused ventricular deviation. Ventricular ERP refers to the longest S1–S2 interval that cannot cause ventricular deflection.

The ventricular arrhythmia (VA) inducibility was measured by the window of vulnerability (WOV). If VA was induced by decremental S1–S2 stimulation, shortest and longest intervals were determined and difference between them was defined as WOV.

### Construction of APD Restitution Curves

The recovery curve of APD was drawn by APD_90_ induced by S2 and corresponding diastolic interval (DI). APD_90_ refers to 90% repolarization duration. DI refers to the time interval from last APD_90_ point to next AP starting point, measured by APD_90_ of S1 subtracted from a very short S1–S2 interval. Then restitution curves were constructed by plotting APD_90_ of S2 against DI. The curves were fitted using a mono-exponential equation (y = y_0_ + A1 [1 - e^–DI/τ^ ^1^]), and maximal slope was calculated by the following equation: (A/τ_1_) × [Exp(-DI/τ_1_)]. The slope of shortest DI refers to maximal slope (S_max_) of the curve.

### Real-Time Polymerase Chain Reaction Analysis

The primers were designed for Rgs5, Tgfβ1, Col1α1, and Col3α1 using Primer 5.0 designing tool. Green qPCR Mix (Aidlab) was used to perform real-time polymerase chain reaction (PCR) with a real-time PCR system (ABI StepOnePlus). The forward and reverse primers for Rgs5, Tgfβ1, Col1α1, and Col3α1 were as follows:

**Table:** 

Rgs5	CACAAACATAGGCAAACCACAG
	TACAAAGCAGTCAGAAAGAACCA
COL1a	CCTCCCAGAACATCACCTATCA
	GGTCTTGGTGGTTTTGTATTCG
COL3a	ATGACTGTCCCACGTAAGCACT
	GGTATGTAATGTTCTGGGAGGC
TGFβ1	CCGCAACAACGCCATCTA
	TCCGTCTCCTTGGTTCAGC
Actin	CTGAGAGGGAAATCGTGCGT
	CCACAGGATTCCATACCCAAGA

### Picro-Sirius Red Staining Assays

The left and right ventricles and atrial appendages were observed by transverse incision near apex. Cardiac sections (4–5 μm thick) stained with Picro-Sirius Red (PSR) were used for collagen deposition. Adobe Photoshop 7.0 software was used to analyze the number of yellow (tissue) and red (collagen) pixels and calculate the percentage of fibrosis (red pixels/[red—yellow pixels]).

### Statistical Analysis

All data were expressed as mean ± standard error of mean. SPSS 16.0 was used to complete statistical analysis performed using Student’s *t* test. *P* < 0.05 was considered statistically significant. Origin 6.0 (MICRO Co., United States) was used to analyze APD recovery curve for non-linear curve fitting.

## Results

### Determination of Structure Remodeling in Ventricle

Echocardiography was applied to confirm whether the electrical alterations were associated with ventricular dilation, which showed that by measuring FS and left ventricular EF, Rgs5^–/–^ mice did not exhibit ventricular dysfunction (Table 1).

**TABLE 1:** Measurement of echocardiographic parameters between WT and Rgs5^–/–^ mice.

	WT (*n* = 6)	Rgs5^–/–^ (*n* = 6)	*P*
LVEDD (mm)	3.58 ± 0.07	3.55 ± 0.08	0.49
LVESD (mm)	2.10 ± 0.09	2.02 ± 0.08	0.11
LVEF (%)	78.83 ± 1.72	80.17 ± 1.60	0.20
FS (%)	42.67 ± 1.37	41.33 ± 1.37	0.12

**FIGURE 1:**
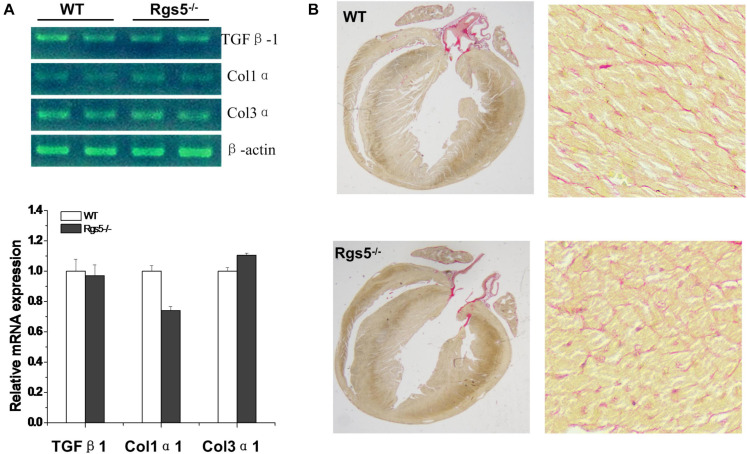
Fibrosis assessment of Rgs5^–/–^ and WT ventricles. The mRNA expression levels of fibrotic mediators including Tgfb1, Col1a1, and Col3a1 were analyzed in the ventricles of WT and Rgs5^–/–^ mice **(A)**. PSR staining was performed on the histological sections of the ventricles in WT and Rgs5^–/–^ mice **(B)**.

Morphologically, by calculating the results of PSR stained sections, the relative areas of WT (*n* = 6) and Rgs5^–/–^(*n* = 6) ventricular fibrosis were accounted to 12.1 ± 3.4% and 10.9 ± 2.7%. There was no significant difference between the 2 groups (*P* > 0.05) (Figure 1B). In addition, mRNA expression of fibrosis mediators including Col1α1, Col3α1 and Tgfβ1 showed similar trends between WT and Rgs5^–/–^ ventricle (*P* > 0.05) (Figure 1A). The relative abundance was calculated using WT value with a reference value of 100%. These suggest that Rgs5^–/–^ did not promote ventricular fibrosis and structure remodeling.

### Rgs5^–/–^ Increased QT Variability Is Not Dependent on HRV

**FIGURE 2:**
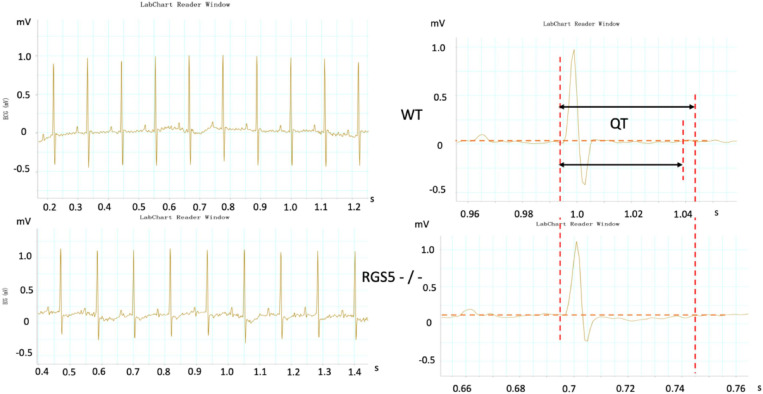
Representative electrocardiograms were recorded in WT and RGS5^–/–^ mice. Results of QT intervals measurement were displayed by waveform.

**FIGURE 3:**
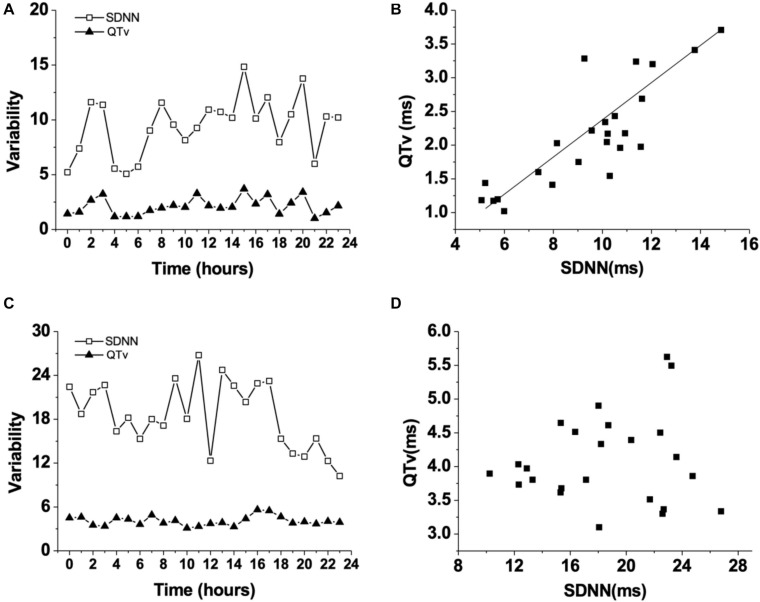
Changes of QT variability (QTv) and SDNN in WT mice during 24 h **(A)**. Correlation between QT variability (QTv) and SDNN in WT mice **(B)**. Changes of QT variability (QTv) and SDNN in Rgs5^–/–^ mice during 24 h **(C)**. Correlation between QT variability (QTv) and SDNN in Rgs5^–/–^ mice **(D)**.

HR, rhythm, and HRV of Rgs5^–/–^ and WT mice were obtained through 24-h telemetry. In 24-h ECG recording, mean RR interval of Rgs5^–/–^ mice did not differ significantly with WT mice (98.4 ± 5.6 vs 100.8 ± 2.1; *P* > 0.05). Moreover, SDNN, SDNNindex, rMSSD, pNN5, and LF/HF of Rgs5^–/–^ mice were higher than WT group (Table 2). Consistent with our previous report (Qin et al., 2012), the QT interval was markedly prolonged in Rgs5^–/–^ mice than in WT mice (57.1 ± 1.8 vs 50.5 ± 0.9; *P* < 0.01) (Figure 2). The variance of QT (QTv) and QTVI over 24 h also increased in Rgs5^–/–^ group (QTv, 22.7 ± 6.3 vs 7.1 ± 2.0; *P* < 0.01; QTVI, -0.71 ± 0.14 vs -0.25 ± 0.06; *P* < 0.01). To determine the correlation between QTv and SDNN, mean values of each hour in a day were analyzed between the two groups (Figure 3). QTv had a positive correlation with SDNN in WT mice (*r* = 0.62; *P* < 0.01), but not in Rgs5^–/–^ mice (*r* = 0.01; *P* > 0.05).

**TABLE 2:** The time-domain and frequency domain parameters analysis of HRV between WT and Rgs5^–/–^ mice.

	Rgs5^–/–^ mice	WT mice	*P*
SDNN (ms)	17.0 ± 5.9	9.1 ± 4.5	0.01
SDNNindex (ms)	12.9 ± 7.9	7.8 ± 4.2	0.03
rMSSD (ms)	13.0 ± 5.9	9.1 ± 4.5	0.04
pNN5 (%)	13.9 ± 3.7	10.8 ± 3.5	0.02
LF/HF	1.0 ± 0.4	0.8 ± 0.2	0.04

### Rgs5^–/–^ Increased Dispersion of Ventricular Repolarization

**FIGURE 4:**
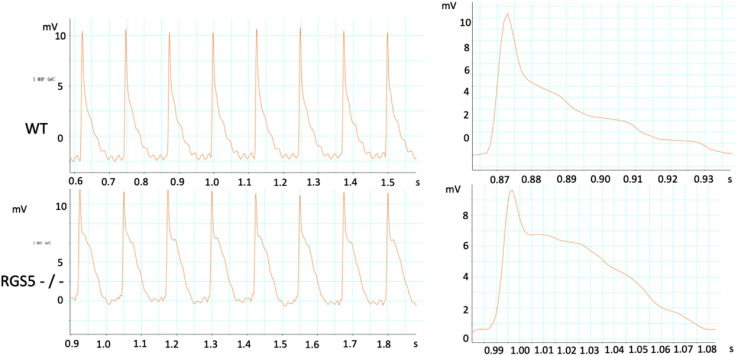
Representative APD was recorded in WT and RGS5^–/–^ mice.

**FIGURE 5:**
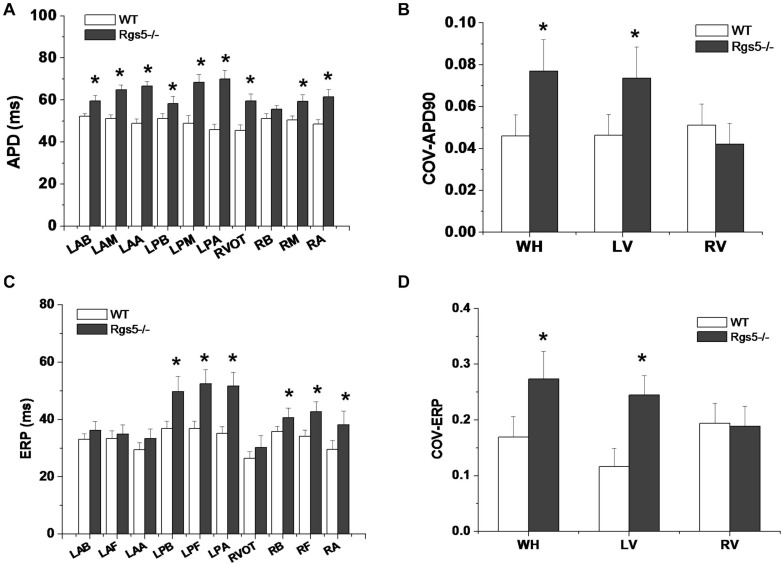
Dispersion of ventricular repolarization in isolated mouse ventricle. Action potential duration (APD) **(A)** and effective refractory period (ERP) **(C)** at different sites of ventricle in WT and Rgs5^–/–^ mice. COV-APD_90_ **(B)** and COV-ERP **(D)** at different sites in WT and Rgs5^–/–^ mice. Left ventricular anterior base (LAB), left ventricle anterior middle wall (LAM), left ventricle posterior base (LPB),left atrial appendage (LAA), left ventricle posterior middle wall(LPM), left ventricular posterior apex(LPA), right ventricular outflow tract(RVOT),right ventricular base(RB), right ventricular middle wall (RM), and right ventricular apex (RA).

The absence of Rgs5 resulted in significant prolongation of APD and ERP in isolated mice ventricles (Figure 4). The increased repolarization was stable at different sites throughout the ventricle. Notably, these alterations showed increased spatial heterogeneity in Rgs5^–/–^ mice, especially in apex and left posterior wall. Thus, the dispersion of epicardial APD_90_ (COV-APD_90_) at sites throughout the whole heart showed a significant difference between WT and Rgs5^–/–^ mice (WT, 0.05 ± 0.01; Rgs5^–/–^, 0.08 ± 0.02; *P* < 0.01) (Figure 5A) and in left ventricle (WT, 0.05 ± 0.01; Rgs5^–/–^, 0.07 ± 0.01; *P* < 0.01) (Figure 5B). Similarly, the dispersion of ERP (COV-ERP) across all heart sites also showed the same trend (WT, 0.17 ± 0.03; Rgs5^–/–^, 0.27 ± 0.05; *P* < 0.01) (Figure 5C) and in left ventricle (WT, 0.12 ± 0.03; Rgs5^–/–^, 0.24 ± 0.03; *P* < 0.01) (Figure 5D).

### Rgs5^–/–^ Steepened APD Restitution Gradient

**FIGURE 6:**
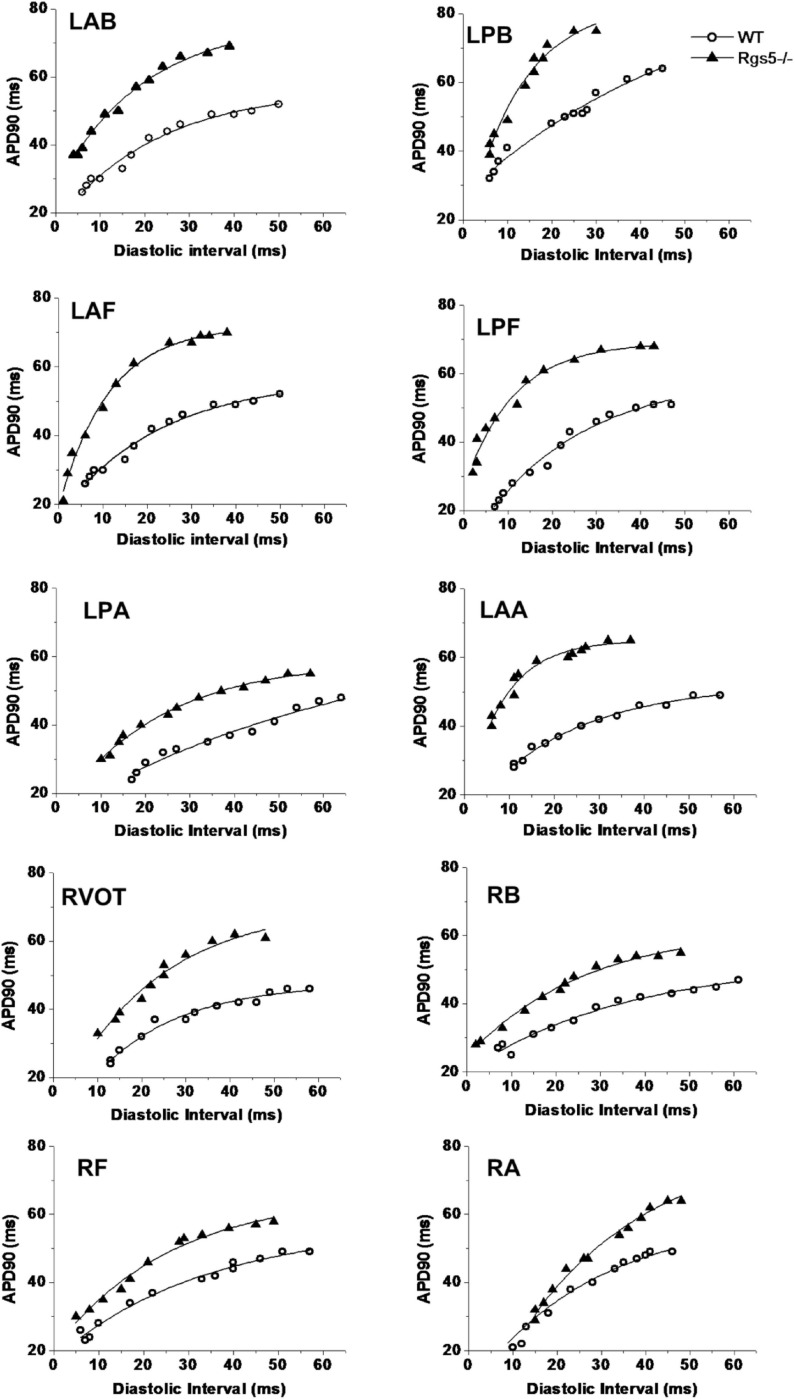
Representative examples of APD restitution curves at each site in WT and Rgs5^–/–^ mice.

**FIGURE 7:**
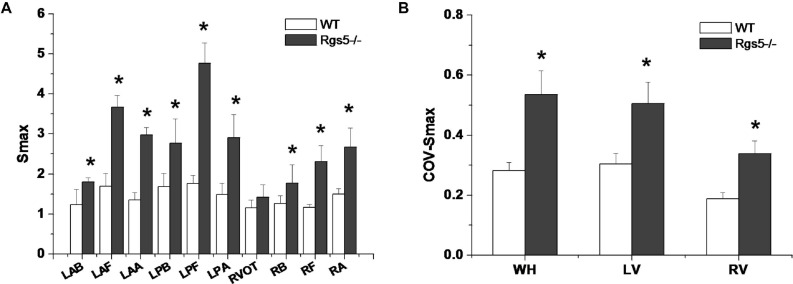
S_max_ **(A)** at different sites of ventricle in WT and Rgs5^–/–^ mice. COV-S_max_ **(B)** at different sites in WT and Rgs5^–/–^ mice.

APD_90_ restitution curves were constructed by S1–S2 pacing method. The absence of Rgs5 markedly steepened the slopes of APD restitution curves at all 10 sites (Figure 6) in the heart (*P* < 0.01) and increased spatial dispersions of S_max_ (COV-S_max_) compared with WT mice throughout whole heart (WT, 0.28 ± 0.03; Rgs5^–/–^, 0.53 ± 0.08, *P* < 0.01). In comparison, the spatial dispersions of S_max_ (COV-S_max_) increased at sites in left ventricle (WT, 0.30 ± 0.03; Rgs5^–/–^, 0.51 ± 0.07; *P* < 0.01) and right ventricle (WT, 0.19 ± 0.02; Rgs5^–/–^, 0.34 ± 0.04; *P* < 0.05) (Figure 7). The increased values of S_max_ were more obvious in left apex and posterior wall, so that COV-S_max_ of left ventricle was larger than that of right ventricle (*P* < 0.01) in Rgs5^–/–^ mice.

### Rgs5^–/–^ Facilitated Ventricular Tachyarrhythmia Inducibility

**FIGURE 8:**
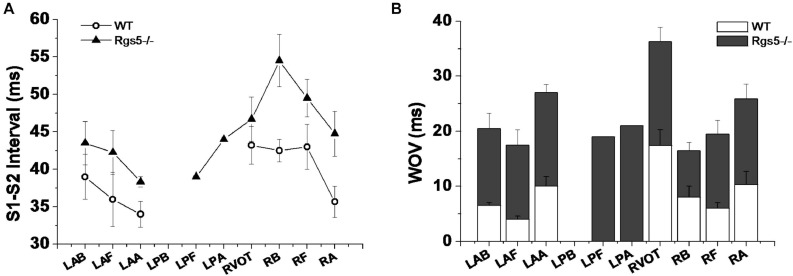
S1–S2 interval-induced ventricular tachyarrhythmia at different sites of ventricle in WT and Rgs5^–/–^ mice **(A)**. Window of vulnerability (WOV) of VA in 10 sites of ventricle in Rgs5^–/–^ and WT hearts **(B)**.

Ventricular tachyarrhythmia was induced by S1–S2 pacing protocol in 5 of 16 hearts (31.2%) in WT group, but in 12 of 16 hearts (75.0%) in Rgs5^–/–^ group. Except that LPB was difficult to induce ventricular arrhythmia, Rgs5^–/–^ greatly increased the induced S1–S2 interval at all sites of heart than that in WT group (*P* < 0.05) (Figure 8A). Moreover, the induction threshold and WOV of VA in 10 sites of ventricle were determined between Rgs5^–/–^ and WT hearts, but VA in left posterior wall was hard to elicit by S1–S2 induction. The results of VA induction threshold analysis showed that WOV of Rgs5^–/–^ heart widened compared with WT heart (*P* < 0.05) (Figure 8).

## Discussion

### Main Findings

The major findings are as follows: (1) Rgs5^–/–^ prolonged ventricular repolarization and increased spatial heterogeneity of repolarization; (2) Rgs5^–/–^ steepened APD restitution curves and increased spatial dispersion among ventricle; (3) ventricular tachyarrhythmia was facilitated by Rgs5^–/–^; and (4) Rgs5^–/–^ induced VA, which was not dependent on myocardial fibrosis.

### Rgs5 and QT Temporal Fluctuation

Several studies found that incidence of arrhythmias is related to the degree of repolarization variability (Thomsen et al., 2004; Lengyel et al., 2007; Hegyi et al., 2017). After interventions resulted in increased repolarization variability, incidence of TdP increased. Although QTc was significantly prolonged, occurrence of TdP did not change (Salem et al., 2016). Therefore, QT variability was considered to be a better predictor of TdP than traditional QT interval assessment. Previously, QT variability has been shown to be elevated in long QT syndrome, ischemia, and congestive heart failure (CHF) (Dobson et al., 2011; Badran et al., 2012; Seethala et al., 2015; Yao et al., 2019). Piccirillo et al. (2009) reported that during CHF, the activation of sympathetic is related to the increase in QT interval and QT variability. These changes may be related to myocardial structural damage and chronic neurohumoral activation, which is characteristic of CHF (Piccirillo et al., 2009). However, no relationship was found between QT variability and sympathetic activation in normal conditions (Arai et al., 2013). This study showed that there was no significant change in cardiac contraction of Rgs5^–/–^ heart compared with WT heart and, importantly, QT temporal change in Rgs5^–/–^ mice was not correlated with HRV. This indicated that Rgs5^–/–^-induced repolarization variability may not be modulated by automatic effects.

In addition, Piccirillo et al. (2009) also conjectured that large reduced repolarization reserve arises from the improper regulation of K^+^ and Ca^2+^ cytosol channels in CHF, which may lead to large fluctuations in QT interval (Salem et al., 2016). Lengyel et al. (2007) also showed that combination of drugs to block I_Kr_ and I_Ks_ can significantly increase QT variability in rabbit model (Dobson et al., 2013). Thus, the reduction of repolarization reserve may contribute to temporal fluctuation of QT interval. Our previous study demonstrated that absence of Rgs5 markedly prolonged cardiac repolarization through attenuated several critical outward K^+^ channels including Kv1.5, Kv2.1, Kv4.2, and Kv4.3. During Rgs5^–/–^-induced repolarization prolongation, there is a poorly compensated ability for these damaged ion channel if necessary.

### Rgs5 and Cardiac Spatial Repolarization

The electrical restitution property of myocardium has been shown to determine susceptibility of the heart to VA. Previous evidences indicated that a steep slope of APD restitution (>1) can promote conduction block and split electrical waves into fibrillation-like state. It was suggested that small changes in DI can lead to a large fluctuation of APD (Shattock et al., 2017). Many previous studies had investigated ionic mechanisms underlying and affecting hysteresis in restitution property. Studies concluded that [Ca^2+^]_i_ dynamics played a key role in APD restitution. During dynamic pacing, Ca^2+^_i_ accumulation increased when APD restitution slope exceeded 1, and slope was flattened by suppressing SR Ca^2+^ cycling with thapsigargin and ryanodine (Goldhaber et al., 2005; Sato et al., 2018). Enhanced I_Kr_ increased the slope of restitution, while the decrease of I_Ca,L_ had effects similar to that of increasing I_Kr_ (Wu and Patwardhan, 2007). However, Decker et al. (2009) found that I_to_ regulates the time course of APD restitution and I_Ks_ plays an important role in shortening of APD with short DIs. As a modulator of multiple repolarizing K^+^ currents, Rgs5^–/–^ can be hypothesized to have a potential effect on APD restitution. In this study, the findings revealed that Rgs5^–/–^ can make APD restitution curve steeper and increase spatial dispersion of S_max_.

Although APD restitution plays an important role in the occurrence of arrhythmia, high induction of cardiac fibrillation cannot be explained by S_max_. This may be because inconsistent compensation promotes greater heterogeneity throughout the heart. Previous studies have shown that high incidence of ventricular tachycardia or ventricular fibrillation is caused by a spatial difference in recovery slopes between different sites of ventricle, and the slope was steeper in apex than base of ventricle (Pak et al., 2004). It has also been reported that vagus nerve stimulation flattens the recovery slope (S_max_ < 1) and promotes atrial fibrillation, which is related to the increased spatial dispersion of S_max_ (Lu et al., 2011). Therefore, APD restitution at a single site of heart cannot represent APD restitution kinetics in entire heart.

The heterogeneity of APD restitution forms repeatable spatial patterns among animals, which is usually attributed to changes of ion channel density in different regions of tissues. Studies have shown that APD was different between guinea pig ventricle and rabbit ventricle, presumably owing to the density and kinetics of components of I_Kr_ and I_K__*s*_ (Lu et al., 2001). London et al. also demonstrated that I_to_ was 30% greater in myocytes from apex than base, which may account for the dispersion of repolarization and refractoriness in mice (London et al., 2007). Consistently, the present study showed APD and ERP were significantly shortened in apex compared with base region in WT mice. As regulator of multiple K^+^ currents, Rgs5^–/–^ disturbed this gradient oriented from base to apex and significantly increases the dispersion of repolarization. Thus, Rgs5^–/–^ seems to be an important determinant of arrhythmia vulnerability.

## Conclusion

The results of this study indicate that Rgs5^–/–^ facilitated ventricular tachyarrhythmia by prolonging ventricular repolarization and increased temporal heterogeneity of repolarization and spatial dispersion in ventricle.

## Data Availability Statement

The original contributions presented in the study are included in the article/supplementary material, further inquiries can be directed to the corresponding author/s.

## Ethics Statement

The animal study was reviewed and approved by the Animal Care and Use Committee of Shanghai Chest Hospital.

## Author Contributions

MQ contributed to the conception of the study and performed the data analysis. ZS performed the experiment and wrote the manuscript. YL performed the data analysis and participated in the writing. XL helped perform the analysis with constructive discussions. All authors contributed to the article and approved the submitted version.

## Conflict of Interest

The authors declare that the research was conducted in the absence of any commercial or financial relationships that could be construed as a potential conflict of interest.
